# Surgical Training in the United Kingdom's National Health Service: The Challenges for International Medical Graduates

**DOI:** 10.7759/cureus.44640

**Published:** 2023-09-04

**Authors:** Farid Gerges, Reda H Mithany, Mark Sidhom, Ioannis N Gerogiannis

**Affiliations:** 1 General Surgery, Kingston Hospital National Health Service (NHS) Foundation Trust, London, GBR; 2 Laparoscopic Colorectal Surgery, Northampton General Hospital, Northampton, GBR; 3 General Surgery, Hampshire Hospitals National Health Service (NHS) Foundation Trust, Basingstoke, GBR

**Keywords:** nhs, international medical graduates, overseas doctors, surgical training, global healthcare system

## Abstract

Background: The UK's National Health Service (NHS) is a hub that trainees from all over the world want to join. However, there are many challenges for International Medical Graduates (IMGs). The aim of this study is to raise awareness of these challenges and to attempt to identify areas for improvement in the surgical training experience for international graduates wishing to join the NHS and obtain a National Training Number (NTN).

Methods: A 33-question survey was designed and distributed to the surgical community via The Upper Gastrointestinal Surgery Society (TUGSS) and social media. Eighty-five respondents, IMGs from 25 countries, participated.

Results: The results showed that 43.5% of doctors had a Master's degree (MSc). Most IMGs joined as locally employed doctors at the senior house officer or registrar level. They all faced many challenges in the UK, including difficulties finding a job in the NHS, obtaining an NTN, and adapting to the differences between UK surgical practice and their home country. More than 50% of doctors did not have a named educational/clinical supervisor, and 63.2% of them felt that the supervisor helped them to become more familiar with the system. The support doctors received from the human resources department of the hospital they joined was poor. In addition, more than half of the IMGs changed their career plans after joining the NHS (56.4%) and would like to stay in the UK (52.9%). The majority of them (43.9%) plan to obtain an NTN.

Conclusions: This study showed that there is a need to support international doctors who wish to start or continue their training in the UK. Furthermore, IMGs should expect to face several challenges when applying to work in the UK NHS.

## Introduction

Surgical training is a challenging endeavour, one that usually requires trainees to explore distant lands in search of the best education available. Among the myriad of options, the United Kingdom is emerging as a favoured destination for honing trainees' skills [1,2]. The UK's renowned National Health Service (NHS) has become a thriving hub for ambitious trainees from all corners of the globe. However, the journey to training in the NHS can present its fair share of obstacles, particularly for overseas surgeons who must navigate cultural and linguistic differences that can pose challenges and barriers along the way [3]. Overseas surgeons wishing to train in the NHS often face the challenges of finding a suitable post in the NHS, obtaining a National Training Number (NTN), and adapting to the differences between surgical practice in the UK and their home country. Competition for training posts can be fierce, and cultural and linguistic differences can affect their ability to navigate the application process effectively. Understanding the requirements and expectations of the NHS, as well as the specific criteria for each training programme, is crucial for prospective trainees [4].

In addition to technical skills, modern surgery emphasises the importance of non-technical skills such as communication, teamwork and decision-making. Overseas surgeons training in the NHS may need to develop these skills to meet the expectations of the healthcare system. Furthermore, academic commitments, research opportunities, and adherence to clinical governance principles are also integral components of surgical training in the UK [5,6].

Therefore, the goal of holistic training in surgery, encompassing both technical and non-technical skills, can be challenging for trainees. The pressure to excel in all aspects of surgical practice can lead trainees to experience stress, discouragement to continue their efforts, and burnout, which can affect their well-being [7-9].

Given that a large number of NHS staff were trained outside the UK, and that approximately 40% of surgeons obtained their primary medical qualification outside the UK, it is important to recognise the particular difficulties faced by overseas surgical trainees [10]. To shed light on these challenges, and to inform stakeholders about possible areas for improvement, a survey was conducted to identify the specific difficulties experienced by overseas surgical trainees in the NHS.

This study aims to identify and analyse the specific challenges faced by international medical graduates (IMGs) in obtaining surgical training in the NHS.

By identifying and raising awareness of these challenges, the study seeks to advocate for the development of support mechanisms and interventions that can improve the surgical training experience for international graduates. These could include targeted training programmes, cultural and language support, and mentoring initiatives to address the specific needs of international surgical trainees.

The results of this survey can serve as a valuable resource for stakeholders involved in surgical training, including educators, policymakers and healthcare leaders. By understanding the challenges faced by international surgical trainees, these stakeholders can make informed decisions to improve the overall training experience and ensure the well-being and success of trainees.

Ultimately, the goal of this study is to promote a more inclusive and supportive environment for overseas surgical trainees in the NHS. By addressing the difficulties they face, we can contribute to their professional development, improve patient care and increase diversity and global perspectives within the UK NHS.

## Materials and methods

This survey was designed to capture a comprehensive understanding of the challenges faced by overseas surgical trainees in the UK. It included questions on various factors such as demographics, qualifications, difficulties encountered, awareness of training requirements, availability of resources, advantages and disadvantages of being an overseas surgeon, recommendations for other surgeons, and future plans.

To ensure the anonymity of respondents, the survey was anonymised, allowing participants to freely express their thoughts and experiences. The survey consisted of 33 questions, including multiple-choice, scale, and short essay questions. This diverse question format aimed to gather a range of responses and perspectives from the participants.

The survey was disseminated globally through The Upper Gastrointestinal Surgery Society (TUGSS) and reached surgical doctors via email and social media platforms. Surgeons were encouraged to share the survey freely, allowing for a broader range of respondents and capturing a more comprehensive understanding of the challenges faced by overseas surgical trainees.

The survey was conducted in English, reflecting the language commonly used in the UK healthcare system. The data collection period ended on November 5, 2022, after which the data was analysed to identify common themes, trends, and challenges faced by overseas surgical trainees in the NHS.

By conducting this survey and collecting data on the difficulties faced by overseas surgical trainees, the study aims to raise awareness about these challenges and provide valuable insights for stakeholders involved in surgical training. The findings from this survey can inform policymakers, educators, and healthcare leaders on potential areas for improvement and support mechanisms that can enhance the training experience for foreign graduates in the UK. In this study, a comprehensive survey consisting of 33 questions was designed by three surgeons from the Kingston Hospital NHS Foundation Trust. The survey was disseminated via TUGSS and was distributed globally to surgical doctors via email and social media. This survey targeted the overseas surgeons who are currently practising or aiming to practice in the UK. Surgeons were invited to share it freely via social media platforms. The survey was designed in English and closed for data analysis on November 5, 2022.

The survey was anonymised and designed to include various questions, including multiple-choice, scale, and short essay questions. This was done to ensure respondents were free to express their thoughts. The variability and choices offered were considered most appropriate by the authors.

This survey aimed to investigate and raise awareness about challenges facing overseas surgical doctors when they apply for or enter surgical training in the UK. Questions were designed to measure various factors. The collected data included basic demographics, such as age, gender, and surgical experience and qualifications before joining the NHS, such as academic achievements. In addition, difficulties encountered when joining the NHS, including the difficulty of finding a job or a training number in the NHS, were included. Finally, some other factors were assessed, such as awareness of requirements for surgical training in the UK, the availability of resources and knowledge about training systems in the UK, advantages and disadvantages of being an overseas surgeon, recommendations for other surgeons joining the NHS, and plans after finishing training.

## Results

**Figure 1:**
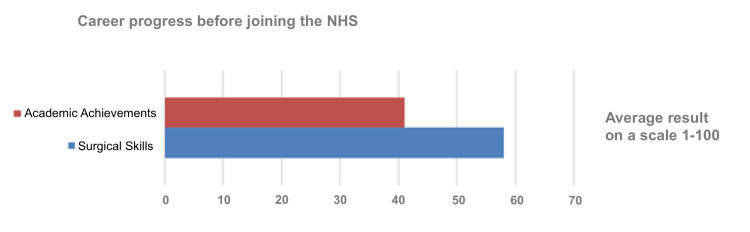
Academic and surgical achievements before joining the NHS NHS: National Health Service

Eighty-five respondents from 25 countries with 12 different mother languages took part in the survey. Of these, 58 (68.24%) were male and 27 (31.67%) were female. On a scale of 1-100, the average number of surgical skills before joining the NHS was 58 and the average number of academic skills was 41 (Figure 1).

Regarding the difficulty of finding a job in the NHS, the average number was 57, and for having an NTN, the average number was 82. This survey revealed that the respondents found a significant difference in practice between the NHS and their home country and on a scale of 1-100, the difference was 64.

**Figure 2:**
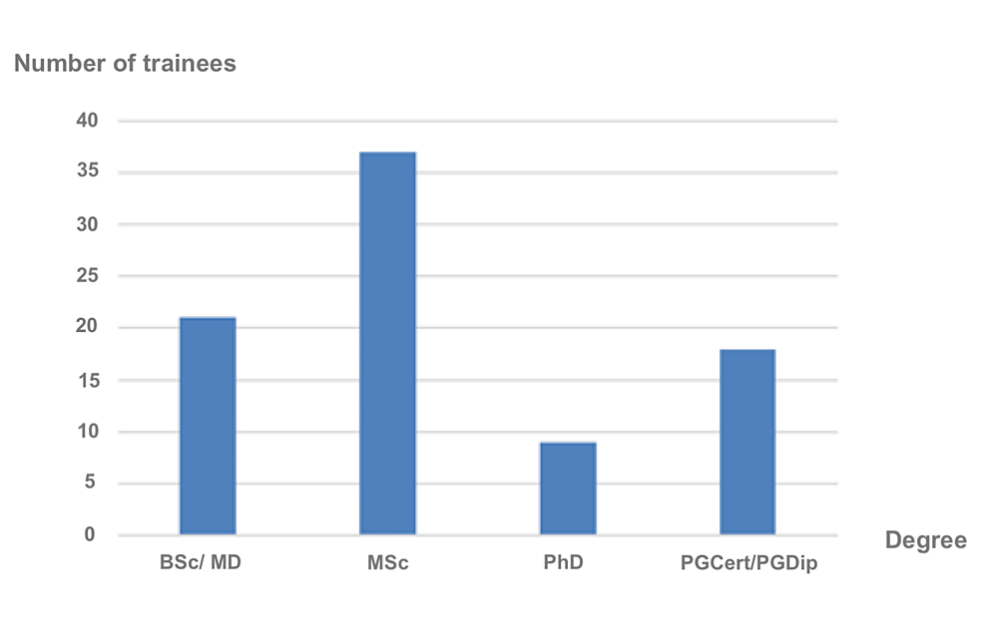
Highest academic degree prior to joining the NHS BSc: Bachelor of Science, MD: Doctor of Medicine, MSc: Master of Science, PhD: Doctor of Philosophy, PGCert: Postgraduate Certificate, PGDip: Postgraduate Diploma, NHS: National Health Service

Twenty-one (25%) of the respondents had only a primary medical qualification such as a Bachelor of Science in Medicine (BSc) or Doctor of Medicine (MD), 37 (43.5%) had a Master of Science (MSc) degree, nine (10.6%) had Doctor of Philosophy (PhD) degree and 18 (20.2%) had a postgraduate certificate (PGCert) or diploma (PGDip) before joining the NHS (Figure 2).

**Figure 3:**
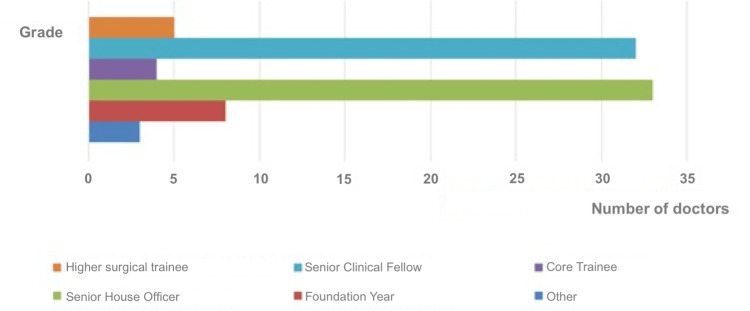
Grade of the International Medical Graduates' first post in the NHS NHS: National Health Service

This survey revealed that eight of the respondents joined the NHS as foundation-year doctors (FYs), 33 as senior house officers (SHOs), and four as core trainees (CTs). Thirty-two as senior clinical fellows (SCFs), five as senior surgical trainees (post-completion of training or associate specialist) and three as other grades (two as Speciality doctors and one as Honorary fellow) (Figure 3).

The average score for clarity of career plan before joining the NHS was 54 out of 100.

**Figure 4:**
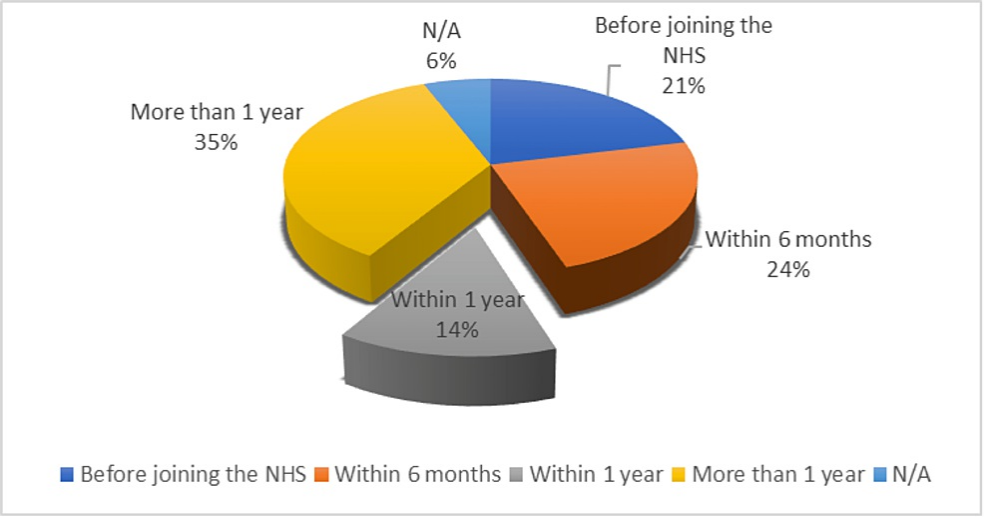
Time spent on career planning NHS: National Health Service

In terms of timing for building a clear idea about a career plan, it was variable. Eighteen (21.2%) respondents had a clear plan before joining the NHS, and 20 (24%) responded within the first six months of joining the NHS. Twelve had a year, and 30 (35%) needed more than a year. Five responses were not applicable (N/A) (Figure 4).

Regarding the guidance and assistance offered to overseas surgeons, 49 respondents were allocated to a named clinical supervisor. Thirty-one of them believed that the supervisor helped them to become more familiar with the system.

**Figure 5:**
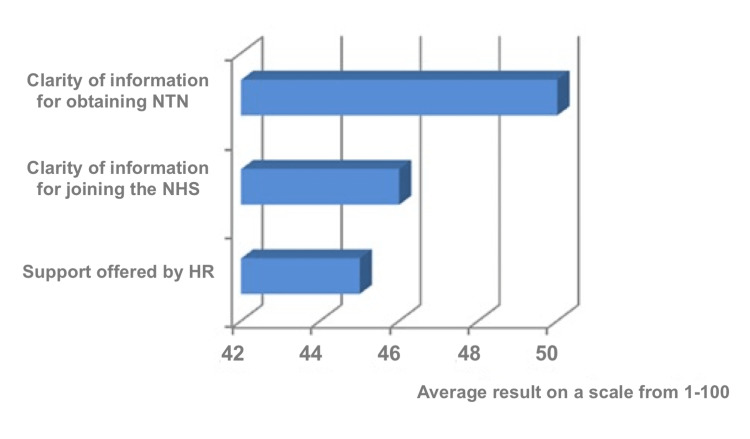
Support and information provided for trainees NTN: National Training Number, HR: Human Resources, NHS: National Health Service

When asked to rate the support offered by the Department of Human Resources (HR), on a scale from 1-100, the average score was 45. Similarly, when rating the clarity of information about working in the NHS and joining official surgical training, the average scores were 46 and 50 respectively (Figure 5).

**Figure 6:**
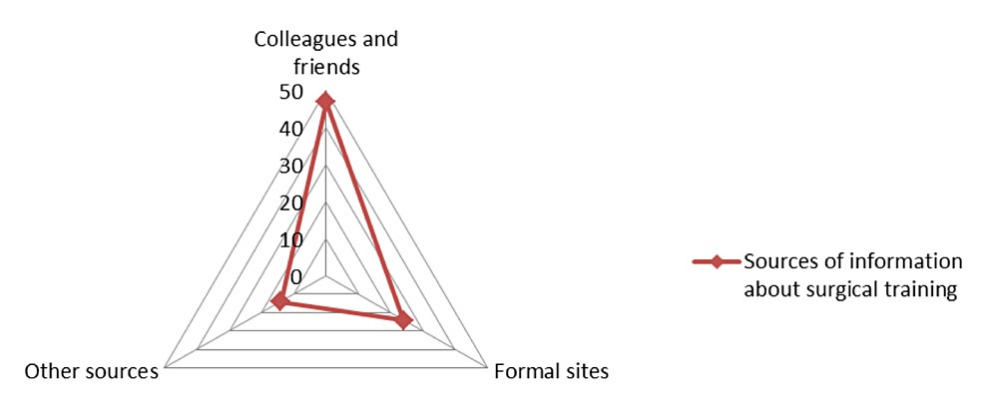
Sources of information for surgical training

In relation to the sources of information available for joining official surgical training, 47 respondents referred to colleagues and friends, 24 used formal sites, and 14 listed other sources such as social media, recruiting agencies, and random searches (Figure 6).

**Figure 7:**
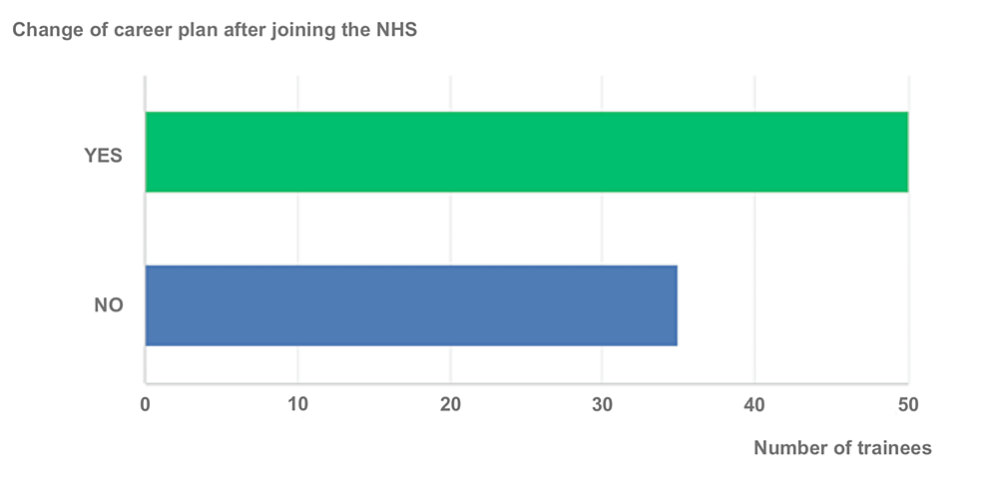
Changing career plans after joining the NHS NHS: National Health Service

The survey also revealed that 50 of the 85 respondents changed their career plans after joining the NHS (Figure 7).

Regarding the difference between the health care system in the UK and the country of origin, overseas trainees highlighted many differences. It can be summarised for the following domains in Table 1.

**Table 1:** Differences, identified by the doctors, between the UK Health System and the Health System in the trainees’ countries of origin NHS: National Health Service

Differences in Healthcare Systems UK vs. Country of Origin	Number of Comments
Health care system and standardisation:	73
Different Policies and logistics	
Availability of local protocols and guidelines in the UK	
Standardisation of medical practice and a more organised working environment	
More available resources, more use of technology in health care systems	
Availability of the primary health services (General practitioner)	
Structured training system which facilitates surgical practice in the UK	
Professionalism and more teamwork in the UK	
More specialised nurses’ roles	
Less patients to see but more paper and admin work	
Different responsibilities, more independent in the UK	
More bureaucratic, transparency and reliability	
More advanced treatment alternatives in the UK	
Less waiting lists	
NHS gets support from government and other organisations such as Royal College of Surgeons (RCS)	
More medico-legal responsibilities	
More specialised units	
Consultant delivered (UK) versus consultant led service (home country)	
More access to equipment, less assistants in theatres	
Culture and variation:	1
Barriers of culture and language difference	
Communication:	23
More patient-centred communication and ethical practices.	
Differences in the consenting process and patients’ confidentiality	
Methods of interaction with patients	
Difference Information technology (IT) and data storage:	10
Use of computer system and electronic recording system	
Training and clinical life:	42
Consultant delivered (UK) versus consultant led service (home country)	
Juniors not well involved in clinical work in the UK (Theatres & clinics)	
Less working hours decreases surgical exposure	
UK has significantly longer training program than other countries	
More controlled juniors’ training in the UK	
Structured training system which facilitates surgical practice in the UK	
Difficulties to get a national training number (NTN) and fewer training opportunities in the UK	
Clinical differences	
Availability of local protocols and guidelines. More evidence-based practices	
More minimal invasive surgeries	
Well-established logbooks	
Enhancement of non-technical skills such as leadership	
Less support from other specialities in the UK	
Slower career progress.	
More practice in the UK than home country	
Academic, audit, research and publication are more than the clinical training	
Investigation-based diagnosis rather than a clinical picture	
Wellbeing:	15
A more stressful working environment in the UK. More stress regarding academic requirements (Audits and research)	
More doctors in the UK	
More free time for family in the UK	
Better salaries and additional payment for emergency shifts.	
Working hours and length of on-calls	
Better work-life balance in the UK	
Lot of mandatory and non-essential work required which ends up with bringing work back home	
Audits and research work:	4
Better chances to get more involved in academic duties	
More Research work and audits	

Responses were divided into the following main domains regarding the challenges of getting a national training number. Multiple challenges were highlighted by trainees in Table 2.

**Table 2:** Challenges to obtain a national training number according to trainees

Challenges Getting a National Training Number (NTN)	Number of Comments Received
Year of graduation got me a high N number which made me need numerous audits, publications and presentations	13
Being a scoring system	1
Exams	3
Travelling/sponsorship requirements and VISA	12
Interview	6
Need for publications	5
Difficult portfolio and lack of time	13
Few opportunities	4
Lack of awareness	2
Lack of guidance/support	9
Inequality and Bias	4
No interest to join training program	2
Being too experienced/Age factor	6
Signing the Certificate of Readiness to Enter Speciality Training (CREST) form	1
COVID effect on health and progress	3
Difficulty to provide adequate evidence	1
Difference in systems	3
Additional degree required	1
Application was not available to overseas when I moved	2
Change in requirements	3
High competition	5
None	1
N/A	18

**Figure 8:**
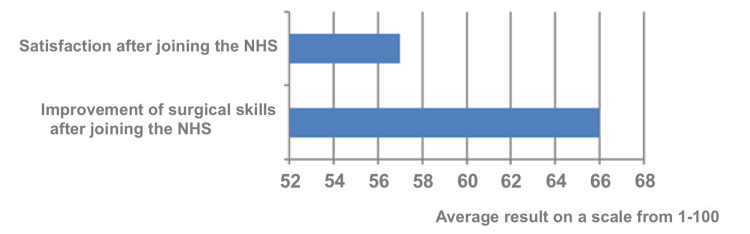
Trainees' reflections on their satisfaction and skills after joining the NHS, using a scale from 1 to 100. NHS: National Health Service

Trainees' responses on future plans were assessed using a one-to-one hundred rating scale. The average score of 57 indicated how well their expectations of joining NHS aligned with their future plans. Moreover, trainees recorded an average score of 66 in regard to the enhancement of their surgical skills after joining the NHS (Figure 8).

**Figure 9:**
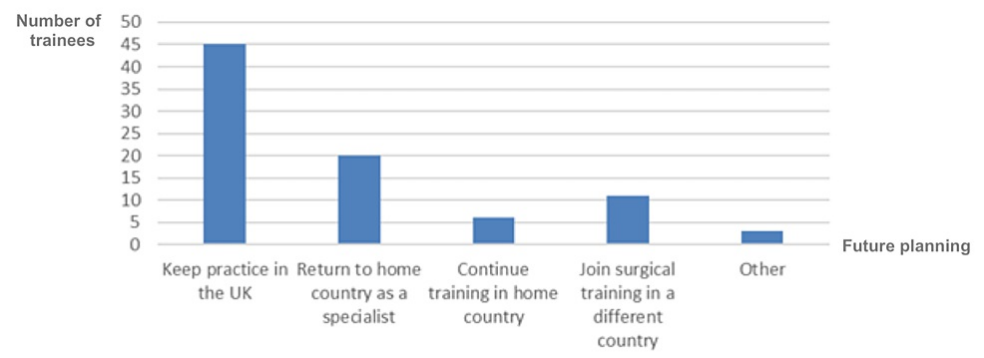
International Medical Graduates' future plans after joining the NHS NHS: National Health Service

When considering their long-term plans, most respondents, comprising 45 individuals (52.9%), expressed their intention of continuing their practice in the UK. Twenty respondents (23.5%) expressed their desire to return to their home country as specialists. Six respondents (7%) aimed to go back to their home country for further training. In contrast, 11 respondents (12.9%) expressed their interest in joining surgical training programs in a different country. Finally, three respondents (3.5%) chose 'others' as their preference, with two aiming to work both in the UK and their home country (Figure 9).

**Figure 10:**
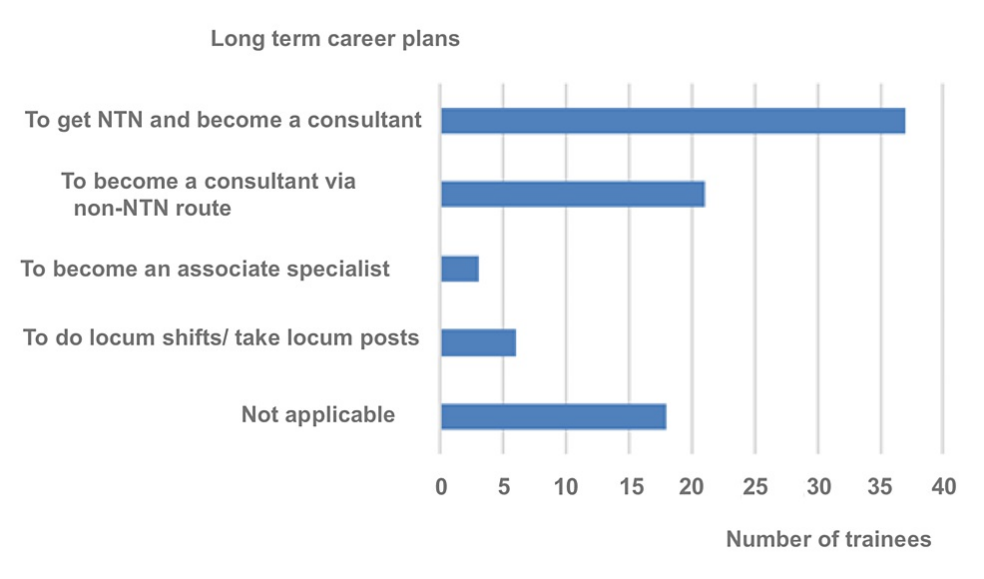
Doctors' plans in the event of a decision to stay in the UK long-term. NTN: National Training Number

In terms of continuing to practice in the UK, the participants' future plans were as follows: 43.5% of the participants plan to acquire an NTN in Surgery to become a Consultant. Around, 24.7% of the participants aim to secure Locally Employed Doctors (LED) jobs and apply through a non-NTN route to become consultants eventually. A total of 3.5% of the respondents are contemplating practising surgery as associate specialists. Seven per cent of the respondents plan to apply for locum positions, and 21.2% responded with 'Not Applicable' (Figure 10).

## Discussion

The field of surgery is undeniably fascinating and full of exciting opportunities. However, it is often accompanied by an inadequate work-life balance, creating a complex and demanding situation. As a result, the field of surgical training has always been a formidable challenge for both educators and trainee surgeons. In order to overcome the hurdles faced by young trainees, numerous international organisations have been set up to address these very obstacles [10,11].

IMGs play an exceptional and essential role in the UK medical community. Recent data from 2022 shows that over 10,000 IMGs have been welcomed into the UK medical register. However, it is concerning to note that IMGs also have a significant exodus rate from the UK workforce. As a result, the General Medical Council (GMC) has called on employers to conduct thorough research into the underlying factors that contribute to IMG attrition [12].

This survey was designed to include all IMGs, regardless of their background or experience, and to collect data on a range of factors that may affect their experience in the UK. Starting a new job is a challenging experience for most IMGs. This can affect their performance and career progression [13]. Therefore, this survey was designed to raise awareness and provide a comprehensive understanding of the challenges faced by overseas surgeons when entering the NHS.

The results of this survey highlight the challenges faced by overseas surgical trainees in the UK, including difficulties in securing a job in the NHS, obtaining an NTN, and adapting to differences in surgical practice between the UK and their home country. This aspect received an average score of 64, suggesting that overseas surgical trainees need assistance in familiarising themselves with the specific protocols, procedures and cultural variations of surgical practice in the UK.

Obtaining an NTN was identified as a significant challenge, with an average score of 82 on a scale of 1-100. Finding a job in the NHS was also identified as a significant challenge, with an average score of 57 on a scale of 1-100. This suggests that overseas surgical trainees often face barriers to securing employment opportunities and progressing in their surgical training.

Hashim A identified many barriers for IMGs in understanding healthcare systems [14]. Slowther et al. highlighted the difficulties faced by IMGs during their transition to practice in the UK [15]. Our survey found that many respondents faced a lack of knowledge resources, with an average score of 46 on a scale of 1-100 for clarity of information about working and joining the NHS, and an average score of 50 on a scale of 1-100 for clarity of requirements to enter formal surgical training. This suggests that IMGs struggle to find comprehensive and accessible information about the requirements and processes involved in working within the UK healthcare system. In addition, the guidance and support offered to overseas surgeons was inadequate. All of this led most of them to change their career plans after joining the NHS. These significant findings highlight the urgent need for more support and resources for overseas surgical trainees. Improving the availability and clarity of information, as well as providing comprehensive guidance and support, can help alleviate the difficulties faced by IMGs and enhance their professional development within the NHS.

Pemberton et al. suggested a number of immigration policies that should be implemented to facilitate the settlement of IMGs and their families [16]. These policies can help to create a more welcoming and supportive environment for IMGs as they transition into the UK healthcare system. Institutions should provide induction and follow-up courses for IMGs to learn about the healthcare system, cultural values and differences, and linguistic subtleties [16]. Equipping IMGs with this knowledge will enable them to better navigate the complexities of the UK healthcare system and provide culturally sensitive care to patients.

From the insights gathered from the survey responses, it is clear that IMGs face many challenges from the time they decide to explore the options for training in the UK to the time they actually join the UK NHS. In an attempt to help address these challenges effectively, we recommend a number of actions or plans. These relate to the different levels of governance.

The first regulatory body an IMG comes into contact with is the GMC. Our recommendations are that the GMC would be wise to:

i) Facilitate the registration process by providing more support and further simplifying materials to help IMGs settle in the UK. This could focus on the issues raised in this study, particularly those relating to the differences between the UK NHS and the applicant's country's healthcare system.

ii) Hold more frequent induction sessions, such as 'Welcome to the UK', after IMGs have registered with it. These sessions should provide further insight into working in the UK and what is expected of IMGs.

iii) Provide training courses tailored to the needs of IMGs at a reasonable cost. This will ensure that they are kept up to date with the latest medical practice, taking into account any financial difficulties they may face.

Immigration and visa services are a crucial and increasingly time-consuming step for IMGs in their transition and settlement in the UK. In an endeavour to make this process as friendly and smooth as possible for applicants, it would be prudent to:

i) Provide clear and concise information about the visa application process, including requirements, realistic timescales and documentation required.

ii) Provide support and guidance throughout the visa application process, addressing any concerns or queries that IMGs may have.

iii) Streamlining the visa application process to minimise delays and ensure timely approval.

iv) Liaise with other organisations, such as HR and NHS hospitals, to coordinate visa requirements and facilitate the smooth integration of IMGs into the healthcare system.

v) Provide resources and information on immigration policies, rights and responsibilities to help IMGs understand and navigate the immigration system effectively.

vi) Establish a dedicated helpline or support service to assist IMGs with any immigration-related issues they may encounter during their time in the UK.

vii) Regularly review and update immigration policies to address any challenges or barriers faced by IMGs and to ensure a fair and efficient immigration process for all.

In our survey, IMGs highlighted the difficulties they encountered with HR in the NHS hospitals they joined. Based on these findings, we recommend that:

i) IMGs need a comprehensive and informative induction, supported by visual materials, to help them understand and prepare for their first job. This should include information on cultural and linguistic differences within the hospital and community.

ii) Financial and psychological support should be provided during the transition period to help IMGs settle in.

iii) Sufficient paid shadowing time should be provided to help IMGs become familiar with the new environment and make effective use of their clinical skills.

iv) IMGs should be directly assigned to clinical and educational supervisors from the outset. These supervisors should guide them in setting career plans and goals, clarifying expectations and responsibilities, and providing information on remuneration, leave, codes of conduct and escalation procedures for concerns.

v) HR would ideally focus more on the well-being of IMGs, ensuring that they have access to the support they need and know how to report harassment or bullying. There should also be greater awareness of their right to speak up and the availability of whistleblowing mechanisms.

vi) In terms of remuneration, IMGs' overseas experience should be fairly taken into account when determining the pay scale and nodal points.

Finally, Health Education England (HEE) is the regulator of national training recruitment. Therefore, HEE should be able to:

i) Provide IMGs with courses and materials to prepare them for training programmes, ensuring that these resources are universally accessible and also highlighting any potential differences with the training system in their country of origin.

ii) Give them as many incentives as possible to stay in the UK and apply for specialist posts after training, rather than leaving to return to their country of origin.

By implementing these recommendations, authorities can better support IMGs, address the challenges they face, and reduce the stress and uncertainty they often experience. This would allow them to focus on their professional roles and contribute effectively to the healthcare system, ultimately improving the healthcare system as a whole.

Our study, being based on a questionnaire survey, has some limitations. Although we tried to disseminate the questionnaire to a global population through social media and a surgical society with a large membership, there is always the presence of sampling bias. In addition, the interpretation of questions may vary between respondents, which is another limitation of a questionnaire survey. Nevertheless, in our study, we tried to create an easily understandable pool of questions that any trainee from any social, economic, religious or other background could understand and not misinterpret.

We tried to make the questions good enough to give us as much information as possible about trainees' experiences and well-being. However, our study did not explore in-depth aspects such as the trainees' experience in theatres, clinics or other clinical duties and whether or not this was influenced by having an NTN. This limitation should be acknowledged, as it could potentially explore clinical experiences and well-being overseas in-depth and provide valuable insights into the impact of having an NTN on practical training and clinical exposure. Future research could consider exploring these aspects to gain a more comprehensive understanding of the issue.

The strength of our study lies primarily in the fact that it is the first to really analyse in depth the challenges faced by IMGs in joining the UK NHS and to make some recommendations on how these can be addressed by national stakeholders and training organisations.

## Conclusions

Overall, the importance of providing adequate support, resources and guidance to IMGs and their families is highlighted by these recommendations and messages from the survey. By addressing these needs, the UK healthcare system can benefit from the skills and expertise that IMGs bring.

The results of this survey shed light on the many challenges that surgical IMGs face when they enter the UK healthcare system when they apply for a national training programme, or when they progress in their surgical careers. These challenges add to the already demanding nature of the surgical profession itself. This survey highlights the need for support for overseas surgical trainees.

Another key aspect highlighted by the survey is the need to increase trainees' awareness of the challenges they will face when applying to the UK NHS. By providing comprehensive information and guidance, trainees can be better prepared for the difficulties they may face and navigate the system more effectively.

In conclusion, this survey highlights the need to support and improve the training and career progression of surgical IMGs in the UK. The GMC, immigration and visa authorities, hospital HRs departments and HEE can all make important steps and changes to support and raise awareness of the challenges faced by IMGs. By addressing these challenges and exploring ways to mitigate them, the NHS can attract and retain more talented overseas surgical trainees, ultimately benefiting patients and the healthcare system as a whole.
